# Outcomes After Chevron Osteotomy with and Without Additional Akin Osteotomy: A Retrospective Comparative Study

**DOI:** 10.1007/s43465-023-00851-4

**Published:** 2023-03-01

**Authors:** Patryk Kuliński, Michał Rutkowski, Łukasz Tomczyk, Grzegorz Miękisiak, Piotr Morasiewicz

**Affiliations:** 1 Department of Trauma and Orthopaedic Surgery, T. Marciniak Lower Silesia Specialist Hospital - Emergency Medicine Center, Fieldorfa 2, 54-049 Wroclaw, Poland; 2 Department of Food Safety and Quality Management, Poznan University of Life Sciences, Wojska Polskiego 28, 60-637 Poznan, Poland; 3 Institute of Medical Sciences, University of Opole, Witosa 26, 45-401 Opole, Poland; 4 Department of Orthopaedic and Trauma Surgery, Institute of Medical Sciences, University of Opole, Witosa 26, 45-401 Opole, Poland

**Keywords:** Radiographic, Hallux valgus, Adjacent-joint arthritis, Chevron osteotomy, Akin osteotomy, Forefoot

## Abstract

**Background:**

Chevron osteotomy is one of the most common approaches to hallux valgus corrective surgery. This procedure is often combined with Akin osteotomy of the proximal phalanx of the hallux. There are no definitive guidelines specifying the indications for a given osteotomy technique nor data on postoperative loss of correction or the effect of the type of first-ray surgery on the development of adjacent-joint arthritis. The aim of this study was to assess radiographic treatment outcomes via chevron osteotomy with and without Akin osteotomy.

**Methods:**

The study evaluated 117 patients treated in the period 2016–2019. Ninety-nine of those patients underwent distal chevron osteotomy alone, and 18 patients underwent a combined chevron–Akin double osteotomy. The analyzed radiograms had been obtained preoperatively, at 6 weeks after surgery, and after a long-term follow-up. The following parameters were assessed: the intermetatarsal angle (IMA), hallux valgus angle (HVA), interphalangeal angle (IPA), postoperative recurrence of valgus deformity, adjacent-joint arthritis, and complications.

**Results:**

Chevron-Akin osteotomy helped maintain lower HVA and IPA values in long-term follow-up in comparison with those in the patients who underwent chevron osteotomy alone. The chevron osteotomy group showed a significant increase in the mean HVA from 18.37° at the first follow-up visit to 20.81° at the last follow-up visit. There were no differences between the groups in terms of the remaining assessed radiographic parameters. Hallux valgus surgery does not increase adjacent-joint arthritis.

**Conclusion:**

The use of combined chevron-Akin osteotomy does not affect HVA or IMA correction. The combination of chevron and Akin osteotomies reduces the risk of increased HVA and IPA in long-term follow-up. The additional Akin osteotomy does not increase the risk of adjacent-joint arthritis. Combining chevron osteotomy with Akin osteotomy is recommended in hallux valgus deformity correction.

## Introduction

Due to its relatively high prevalence, with 28.4% of the adult population affected in the UK, hallux valgus deformity is an important problem for orthopedic surgeons [1–5]. Hallux valgus deformity affects 23% of patients aged 18–65 years, and 35.7–65% of patients over 65 years old [2, 3]. With over 200,000 hallux valgus correction surgeries performed annually in the US [3] this procedure is one of the most common foot surgeries [1].

There have been many surgical techniques of hallux valgus correction described in the literature. The authors reported satisfactory outcomes with various techniques; therefore, there is no gold standard in the treatment of this deformity, and it is usually the orthopedic surgeon who selects the surgical technique [3, 6, 7]. Out of the over 100 described surgical techniques, one of the most thoroughly established and widely used is chevron osteotomy.

The complication rates following hallux valgus correction surgery range from 1 to 75% [1, 3, 4, 8], with the most common undesirable outcomes including incomplete correction, deformity recurrence, nonunion, overcorrection (hallux varus), malunion, arthritis, and the need for fixation removal [1, 6–12]. Some of the complications produce pain and limit movement in the foot, which results in poor treatment outcomes [1, 8, 12].

Chevron osteotomy is characterized by a number of advantages, such as being simple and less invasive than other techniques and ensuring minimal metatarsal shortening [8, 13–15]. The need for revision surgery following distal metatarsal osteotomy is less common than that following proximal metatarsal osteotomy [16]. Some authors believe that a properly performed chevron osteotomy yields positive outcomes in patients with moderate-to-severe hallux valgus deformity [13, 14, 17, 18]. Other authors reserve distal first metatarsal osteotomy only for mild-to-moderate hallux valgus deformity correction [7, 8, 10, 19, 20]. Mild, moderate, and severe deformities are defined as hallux valgus angle (HVA) values of < 20°, 20–40°, and > 40°, respectively, and as intermetatarsal angle (IMA) values of < 11°, 11–16°, and > 16°, respectively [8].

Apart from the first metatarsal osteotomy and soft-tissue procedures, the treatment is often combined with an osteotomy of the first proximal phalanx, such as Akin osteotomy. The objective of Akin osteotomy—a medial closing-wedge osteotomy—is to preserve the lateral cortex of the proximal phalanx during axis correction [20–22].

The purpose of hallux valgus surgery is to achieve radiographic deformity correction with good functional outcomes, which include improved foot biomechanics and pain alleviation [9, 23–25]. Moreover, hallux valgus deformity in the elderly is associated with a higher risk of falls and, consequently, increased mortality [26].

Performing an optimal hallux valgus correction surgery poses a challenge even for experienced orthopedic surgeons. The use of combined chevron and Akin osteotomy has not been fully explored. There have been only three studies comparing selected radiographic parameters following a combined chevron-Akin osteotomy with those after chevron osteotomy alone [20, 22, 27].

Kaufmann et al. compared the outcomes of HVA and IMA correction in patients with hallux valgus deformity with the use of a combined chevron–Akin double osteotomy and chevron osteotomy alone [19, 22]. Those authors observed better hallux valgus correction in the chevron–Akin group. Lechler et al. reported an improved hallux valgus correction in the chevron–Akin osteotomy group in comparison with that achieved after chevron osteotomy alone [20]. Those authors did not assess the rates of arthritis.

We hypothesized that combining chevron osteotomy with Akin osteotomy would affect the radiographic outcomes in chevron osteotomy patients. The purpose of our study was to assess the radiographic outcomes in patients undergoing chevron osteotomy with and without simultaneous Akin osteotomy.

## Materials and Methods

This retrospective study evaluated the patients who underwent hallux valgus deformity correction via chevron osteotomy in our ward in the years 2016–2019. We stratified the patients into those who did and did not undergo an additional, simultaneous Akin osteotomy of the first proximal phalanx.

The study inclusion criteria included symptomatic hallux valgus deformity (with mild to severe radiographic findings) [8, 28], correctly performed radiographic diagnostics of the foot under weight-bearing conditions, patient age of over 18 years, a follow-up period of at least 3 years, hallux valgus correction via chevron osteotomy alone or combined with Akin osteotomy, and complete medical and radiographic records.

The exclusion criteria were a foot deformity requiring a different additional correction procedure, advanced comorbidities (e.g. neuropathy, diabetic complications, rheumatoid arthritis, peripheral vascular disease) that could affect potential complications, and a history of orthopedic surgery of the foot [1, 29].

**Fig. 1:**
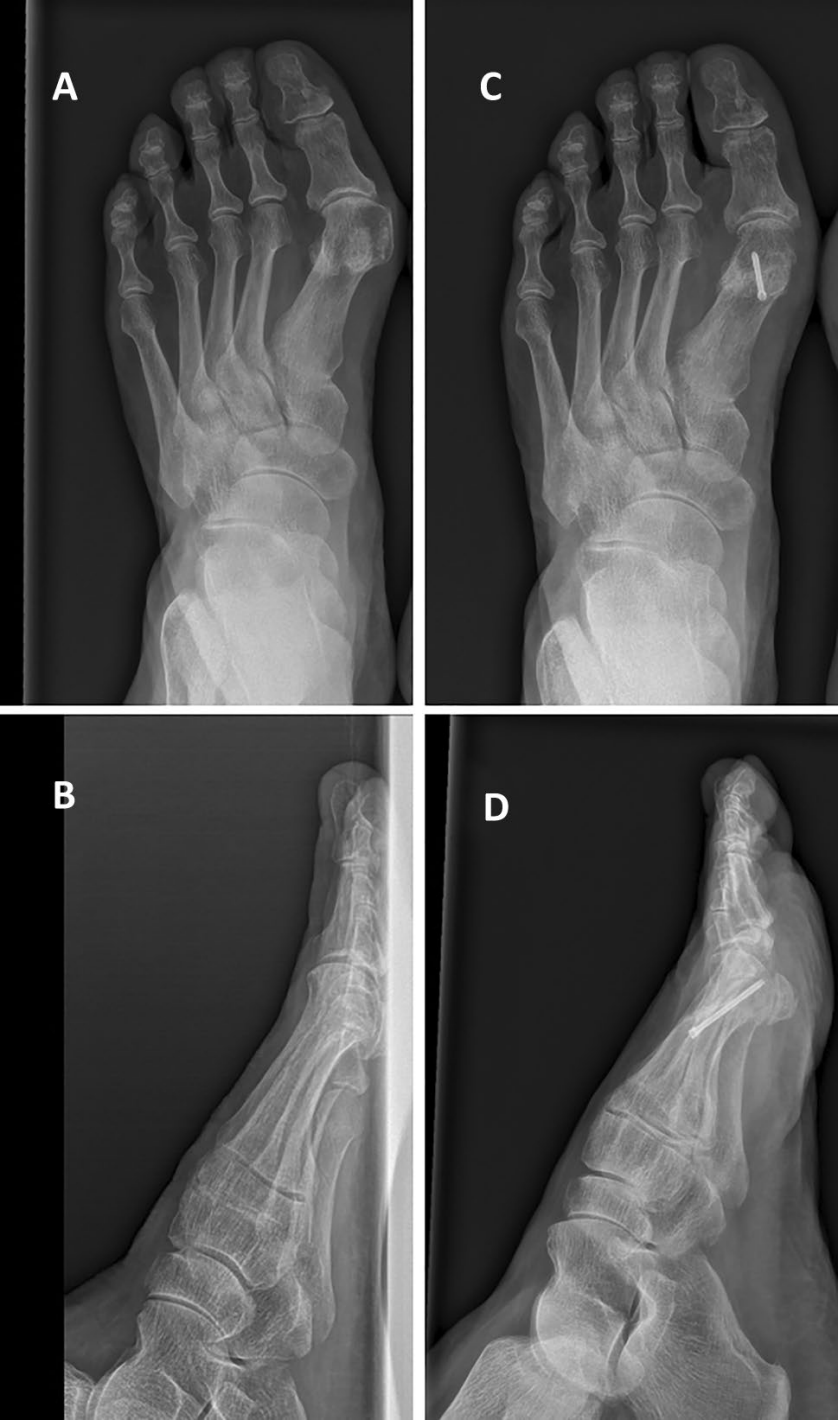
Images of one patient before (**A**, **B**) and after (**C**, **D**) chevron osteotomy

**Fig. 2:**
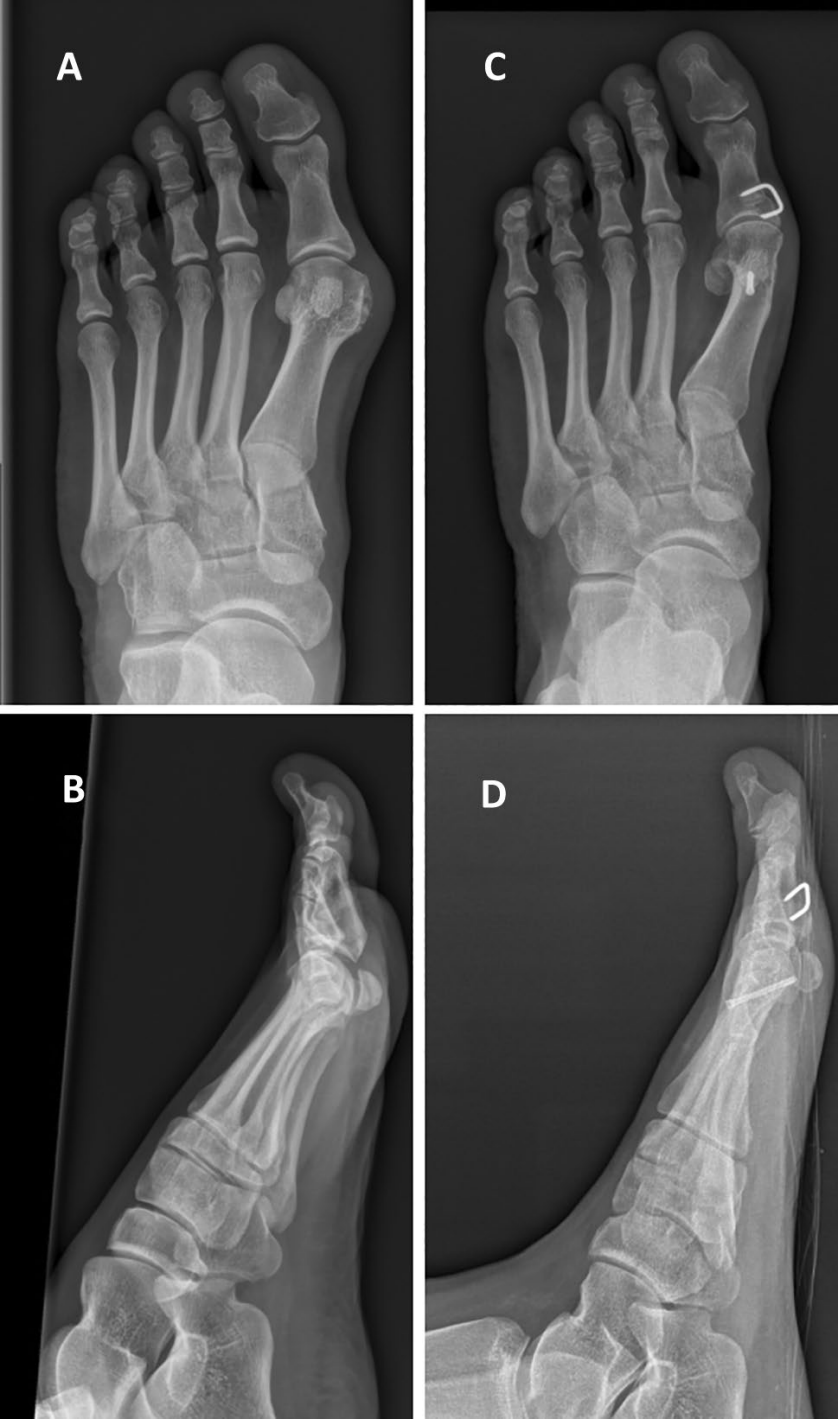
Images of one patient before (**A**, **B**) and after (**C**, **D**) chevron osteotomy combined with Akin osteotomy

Applying the inclusion and exclusion criteria resulted in 117 cases of hallux valgus deformity correction that were eligible for our retrospective evaluation. The procedures had been performed in 110 females (94.02%) and 7 males (5.98%) and included chevron osteotomy alone (99 procedures), Fig. 1 or chevron osteotomy combined with Akin osteotomy of the first proximal phalanx (18 procedures), Fig. 2. If the adequate correction was not achieved with chevron osteotomy, the surgery was expanded to additionally include Akin osteotomy, as suggested in the literature [30, 31].

The mean patient age was 58.91 years in the chevron group and 64.92 years in the chevron–Akin group, with the age difference between the groups not statistically significant (*p* = 0.0778).

All surgeries were performed by one of three experienced surgeons. Intrathecal anesthesia and regional ischemia (achieved with Esmarch tourniquet compression at the level of the thigh) had been used in both types of procedures. Each surgery involved a medial incision directly over the first metatarsal and the metatarsophalangeal (MTP) joint. The incision was extended distally if an additional first proximal phalanx osteotomy was required. An additional dorsal incision was employed for lateral soft tissue release. Subsequently, once the periosteum had been gently scraped off, the bunion was cut off from the first metatarsal and a chevron-shaped cut ranging from 60° to 90° (with the apex at the center of the first metatarsal head) was made with a narrow oscillating saw. The head of the metatarsal was then shifted laterally, and the achieved correction was assessed by simulated weight-bearing and fixed with the use of a Herbert screw or a Kirschner wire. Excess bone on the medial and lateral aspects of the metatarsal head was cut off again, and any osteophytes were removed.

In those patients who required an additional Akin osteotomy of the first proximal phalanx, the initial incision was extended distally. An oscillating saw was used to cut out an adequately sized medial wedge, perpendicular to the long axis of the bone, leaving a lateral cortical hinge intact. Once the bone axis was corrected, fixation with a staple-shaped molded Kirschner wire was employed.

The protocol for medial and lateral release was consistent in all the patients, as it was the one well-established in our ward. Soft-tissue release was conducted before osteotomy. Medial soft-tissue release involved relaxing the taut articular capsule manually through the existing medial incision. Lateral tissue release was conducted via a dorsal incision between the first and second metatarsal heads and involved capsule relaxation and adductor tenotomy.

Following surgery, all patients underwent identical rehabilitation and physiotherapy protocols. A forefoot off-loading orthopedic shoe was recommended for the initial 6 weeks after surgery. Subsequently, normal flat-soled footwear was allowed. After cutaneous suture removal, the patients started their rehabilitation regimen involving physical therapy and manual therapy in an outpatient setting to prevent excessive first MTP joint stiffness and scar pain.

We assessed the duration of hospital stay and the following radiographic parameters: the IMA, HVA, interphalangeal angle (IPA), achieved correction, overcorrection (hallux varus), deformity recurrence, fixation loosening, the need to remove fixation, development of adjacent-joint arthritis, nonunion, and first metatarsal head necrosis.

The IMA was defined as the angle between the line coursing through the centers of the head and base of the first metatarsal bone and the corresponding line coursing along the second metatarsal bone [32–34]. The HVA was defined as the angle between the line passing through the centers of the head and base of the first metatarsal bone and the line passing through the centers of the head and base of the first proximal phalanx [32–34]. Another assessed parameter was the IPA, defined as the angle between the lines passing through the centers of the head and base of the first proximal and first distal phalanx [34, 35].

HVA and IMA values were assessed prior to surgery, at 6 weeks after surgery, and at the last follow-up visit within the study period. The IPA value was assessed prior to surgery and after a long-term follow-up. The achieved correction was assessed based on the difference between the HVA and IMA values measured during the first follow-up visit and the HVA and IMA values obtained before surgery. Maintenance of correction was calculated based on the difference between the angle values measured at the last and first follow-up visits.

Adjacent-joint arthritis was assessed in the first MTP joint, first interphalangeal joint, metatarsal-cuneiform joint, Lisfranc joint, Chopart joint, and in all those joints collectively.

### Statistical Analysis

Data were statistically analyzed using Statistica 13.1. The Shapiro–Wilk test was used to check for normality of distribution. The Student's *t* test was used to compare quantitative variables. For qualitative variables, the Pearson chi-square test was used. The level of statistical significance was set at *p* < 0.05.

## Results

The results have been presented in Tables 1, 2, 3, 4.

**Table 1:** Detailed results of the radiological assessment of individual subgroups

Analyzed variable (mean ± standard deviation)	Chevron group *n* = 99	Chevron + Akin group *n* = 18	*p* value
HVA before surgery [0]	29.9 ± 7.54	29.56 ± 7.5	0.741*
IMA before surgery [0]	11.98 ± 3.12	11.51 ± 3.18	0.124*
Days of hospitalization	2.87 ± 1.82	2.83 ± 1.61	0.214*
I metatarsophalangeal joint arthritis before surgery [%]	90.91	100	0.183**
Interphalangeal joint arthritis before surgery [%]	43.44	61.12	0.166**
Metatarsal cuneiform joint arthritis before surgery [%]	29.3	44.45	0.203**
Lisfranc joint arthritis before surgery [%]	14.15	33.33	0.046**
Chopart joint arthritis before surgery [%]	7.070	5.55	0.814**
HVA last control visit [0]	20.81 ± 7.78	14.7 ± 5.76	0.011*
IMA last control visit [0]	8.73 ± 5.31	9.01 ± 1.11	0.856*
Total arthritis in last control visit [%]	1.36 ± 1.43	1.55 ± 1.42	0.603*
I metatarsophalangeal joint arthritis in last control visit [%]	91.91	100	0.211**
Interphalangeal joint arthritis in last control visit [%]	47.48	66.67	0.134**
Metatarsal cuneiform joint arthritis in last control visit[%]	31.32	50	0.124**
Lisfranc joint arthritis in last control visit [%]	15.16	33.33	0.064**
Chopart joint arthritis in last control visit [%]	8.090	5.55	0.711**
Hardware destabilization [%]	9.090	5.56	0.621**
Nonunion [%]	3.030	0	0.454**
Reoperation [%]	12.120	0	0.709**
HVA first control visit [0]	18.37 ± 6.31	15.17 ± 8.08	0.067*
IMA first control visit [0]	8.11 ± 2.72	8.37 ± 1.69	0.709*
IPA before surgery [0]	5.07 ± 2.38	11.02 ± 4.26	*p* < 0.001*
IPA last control visit [0]	8.6 ± 2.33	6.8 ± 2.16	0.034*
Age of patients [years]	58.59 ± 13.37	64.59 ± 11.88	0.077*

**Pearson's chi-squared test

*Student's *t* test

**Table 2:** Detailed arthritis assessment of patients before surgery and in the last control visit in the Chevron + Akin group and Chevron group

analyzed variable (mean ± standard deviation)	Before surgery	Last control visit	*p* value
Chevron + Akin group			
I metatarso phalangeal joint arthritis [%]	100	100	1**
Interphalangeal joint arthritis [%]	61.11	66.61	0.728**
Metatarsal cuneiform joint arthritis [%]	44.45	50	0.738**
Lisfranc joint arthritis [%]	33.34	33.34	1**
Chopart joint arthritis [%]	5.56	5.56	1**
Chevron group			
I metatarso phalangeal joint arthritis [%]	90.9	91.91	0.799**
Interphalangeal joint arthritis [%]	43.43	47.47	0.568**
Metatarsal cuneiform joint arthritis [%]	29.3	31.32	0.757**
Lisfranc joint arthritis [%]	14.15	14.15	0.840**
Chopart joint arthritis [%]	7.080	8.080	0.788**

**Pearson's chi-squared test

*Student's *t* test

**Table 3:** Achieved correction and recurrence of deformities in the Chevron + Akin group and Chevron group

Analyzed variable (mean ± standard deviation)	Chevron + Akin group	Chevron group	*p* value*
Achieved correction: difference between HVA before surgery and first control visit [0]	15.22 ± 3.96	11.81 ± 4.68	0.102
Achieved correction: difference between IMA before surgery and first control visit [0]	3.61 ± 1.21	4.11 ± 1.32	0.556
Recurrence of deformities: difference between HVA in first control visit and last control visit [0]	4.53 ± 3.51	4.36 ± 4.02	0.955
Recurrence of deformities: difference between IMA in first control visit and last control visit [0]	1.89 ± 2.321	2.57 ± 4.12	0.83

*Student's *t* test

**Table 4:** HVA and IMA values in the Chevron + Akin group and Chevron group

Analyzed variable (mean ± standard deviation)	Before surgery	First control visit	*p* value*
Chevron + Akin group			
HVA [0]	29.90 ± 7.54	15.17 ± 8.08	*p* < 0.001
IMA [0]	11.51 ± 2.31	8.37 ± 3.21	*p* < 0.001
Chevron group			
HVA [0]	29.9 ± 5.54	18.37 ± 5.31	*p* < 0.001
IMA [0]	11.98 ± 2.12	8.11 ± 2.21	*p* < 0.001

Analyzed variable (mean ± standard deviation)	First control visit	Last control visit	*p* value*
Chevron + Akin group			
HVA [0]	15.17 ± 8.08	14.7 ± 5.76	1
IMA [0]	8.37 ± 3.21	9.01 ± 1.11	0.199
Chevron group			
HVA [0]	18.37 ± 5.31	20.81 ± 6.7	0.031
IMA [0]	8.11 ± 2.21	8.73 ± 4.31	1

*Student's *t* test

In the group who underwent chevron osteotomy alone the mean baseline HVA, IMA, and IPA were 29.9°, 11.98°, and 5.07°, respectively. The mean HVA and IMA values of 29.56° and 11.51°, respectively, in the chevron–Akin osteotomy group were similar to those in the previous group; however, the IPA was higher at 11.02°. The difference was statistically significant for IPA values (*p* = 0.0001), unlike for either HVA or IMA values (*p* = 0.7412 and *p* = 0.1241, respectively); Table 1.

The inter-group differences in HVA and IMA values calculated from the measurements taken before surgery and at the first radiographic follow-up were not significant; Table 3. The patients from the distal chevron osteotomy group achieved a mean improvement in HVA by 11.81° and in IMA by 4.11° at the first radiographic follow-up visit, whereas the patients from the combined chevron–Akin osteotomy group achieved a mean improvement by 15.22° and 3.61° (*p* = 0.1026 and *p* = 0.5568), respectively; Table 3.

The study groups showed no significant differences in terms of the rate of recurrence; Table 3. The HVA increased by a mean of 4.36° in the chevron group, whereas in the chevron–Akin group it showed a further decrease by 4.53° (*p* = 0.955). The chevron group exhibited a slightly higher increase in the IMA (by 2.57°) than the chevron–Akin group (by 1.89°) (*p* = 0.83).

**Fig. 3:**
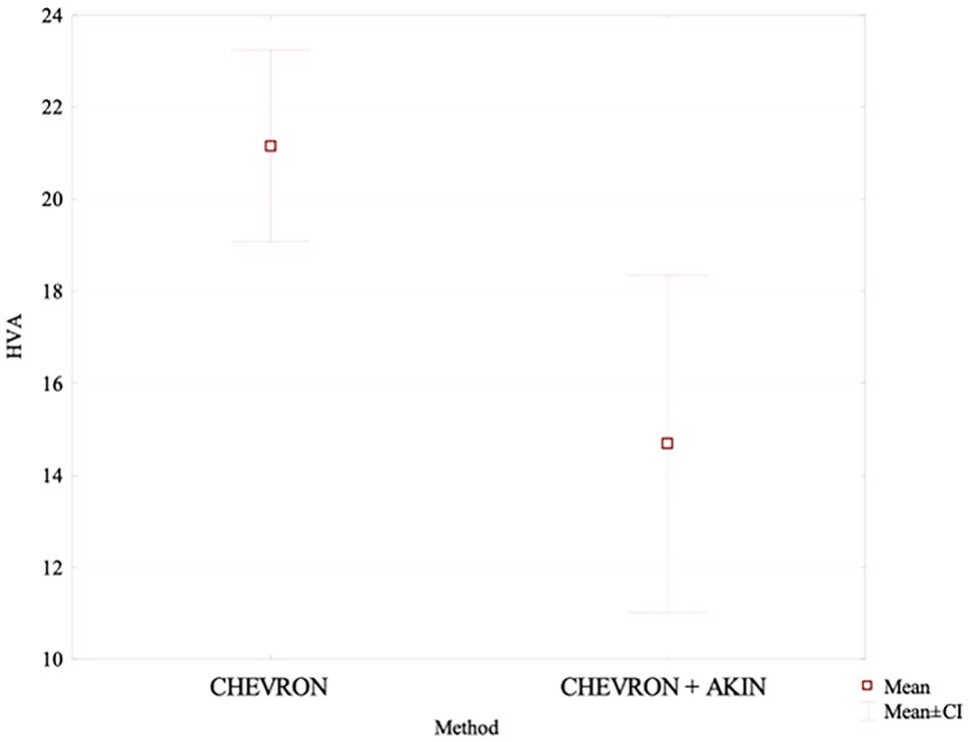
The long-term follow-up HVA value in the chevron-Akin osteotomy group and chevron osteotomy group

**Fig. 4:**
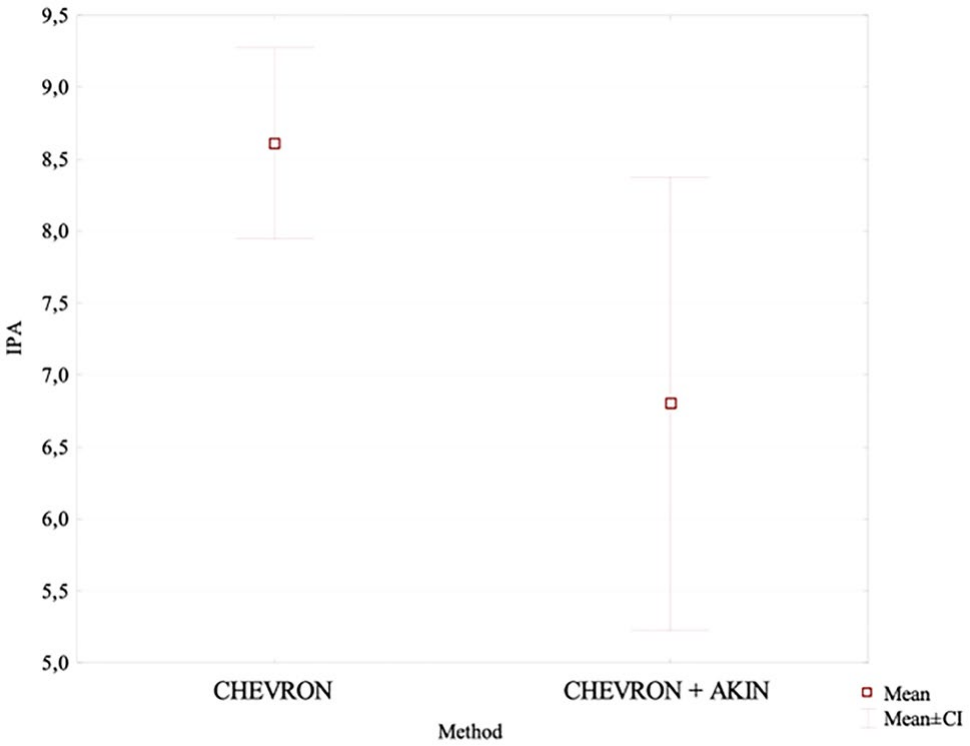
The long-term follow-up IPA value in the chevron-Akin osteotomy group and chevron osteotomy group

**Fig. 5:**
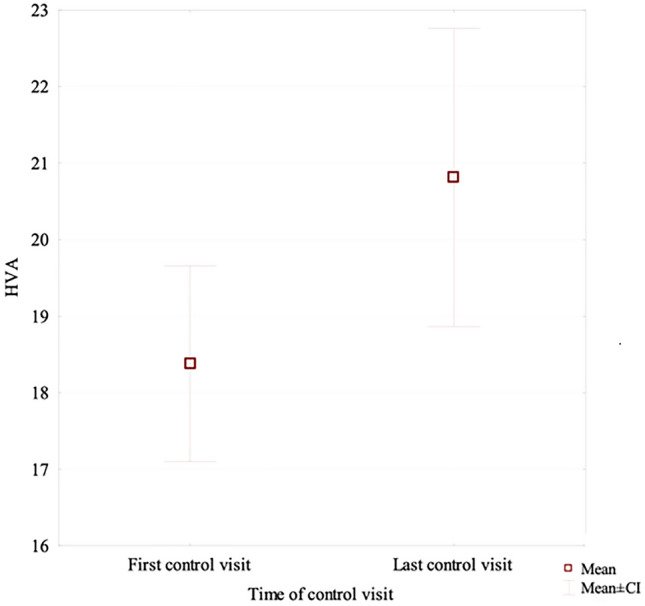
The HVA value at the first follow-up and at the last follow-up visit in the chevron osteotomy group

After a long-term follow-up, the mean HVA value in the chevron-Akin osteotomy group was lower at 14.7° than the value of 20.81° measured in the distal chevron osteotomy group. This was a statistically significant difference (*p* = 0.011), Fig. 3. At the same time, IMA values in the two groups were comparable at 9.01° and 8.73°, respectively. The difference was not statistically significant. After a long-term follow-up, the IPA value in the chevron group was higher at 8.6°, whereas in the chevron–Akin group it showed a further decrease to 6.8°; this difference was statistically significant (*p* = 0.034), Fig. 4. The chevron osteotomy group exhibited a significant increase in the HVA value from 18.37° at the first follow-up visit to 20.81° at the last follow-up visit (*p* = 0.031); Table 4, Fig. 5.

There were no significant differences between the evaluated groups in terms of the rates of loosened fixation, nonunion, or revision surgery; Table 1. No patients from either group developed first metatarsal head necrosis or overcorrection (hallux varus).

Table 2 shows detailed data on the distribution of adjacent-joint arthritis. Neither group showed the rates of arthritis assessed at the last follow-up visit to be greater than those assessed at baseline.

The duration of hospital stay in both groups was comparable at 2.86 days (chevron alone) and 2.83 days (chevron–Akin); Table 1. This difference was not statistically significant (*p* = 0.2144).

## Discussion

This study was to assess how an additional Akin osteotomy would affect various radiographic parameters in patients with hallux valgus deformity treated with chevron osteotomy. The chevron–Akin double osteotomy group showed lower HVA and IPA values at the last follow-up visit in comparison with those measured in the distal chevron osteotomy group. Patients in the chevron group showed an increase in HVA between the first and last postoperative follow-up visits. Our study results support our initial hypothesis.

There is no consensus among foot surgeons as to the superiority of any specific surgical technique in hallux valgus deformity correction [3, 6, 7, 10, 16]. The purpose of hallux valgus correction surgery is to improve the esthetic appearance and function of the foot, ensure its better fit to typical footwear, and alleviate pain [9, 23–25]. Incomplete hallux valgus correction, deformity recurrence, and the risk of complications worsen treatment outcomes.

The use of additional techniques, such as Akin osteotomy of the first distal phalanx, has not been recommended for any specific indications. Stydrom et al. presented the concept of the total valgus deformity of the hallux and calculated that the contribution of the IPA to the total hallux valgus angle was 28.3% [35]. This demonstrated how great an effect the hallux valgus interphalangeus deformity has on the overall hallux valgus deformity. Most authors believe that the use of an additional Akin osteotomy should be at the discretion of the surgeon, and many indicate a number of benefits from using this procedure; however, no authors give any specific guidelines [19, 20, 31, 36]. In one of their articles, Kaufmann et al. recommended combining a chevron osteotomy with an Akin osteotomy when the calculated preoperative proximal-to-distal phalangeal articular angle (PDPAA) is greater than 8° [19]. The PDPPAA determines the relationship between the proximal and distal articular surface of the proximal phalanx. According to the available literature, measurements of the PDPAA—like those of the IPA—are highly repeatable and reliable [37].

The mean baseline deformity in our patient population was similar to that reported by other authors [19, 20, 31, 36]. This is also true for the IPA in the individual study groups, i.e. 5.07° in the chevron group and 11.02° in the chevron–Akin group [20].

Additional use of Akin osteotomy in our study did not help achieve better hallux valgus correction angles in patients with similar baseline HVA and IMA values. Conversely, other authors achieved better correction via additional Akin osteotomy [19, 20, 22].

Kaufmann et al. assessed the effects of an additional Akin osteotomy in patients undergoing chevron osteotomy via a retrospective analysis of 859 feet (785 after chevron osteotomy and 74 after chevron–Akin osteotomy). Those authors observed a significantly greater improvement in the HVA after combined chevron–Akin osteotomy (8.6°) than after chevron osteotomy alone (13.9°). Moreover, they reported an overall greater potential for deformity correction in terms of other evaluable parameters with the use of combined chevron–Akin osteotomy. However, Kaufmann et al. did not analyze complications other than radiographic recurrence (HVA > 20° or IMA > 10°), whose rates were also higher after chevron osteotomy alone (52 cases) [19].

A prospective study conducted by Lechler et al. in 72 patients showed a better achieved HVA correction with the additional use of Akin osteotomy. However, this difference may have resulted from the significantly greater baseline deformity in this group of patients (32° in the chevron–Akin group vs. 27° in the chevron group) [20]. In our study both the mean baseline HVA and the mean baseline IMA values were similar in both groups.

We would like to emphasize that the addition of Akin osteotomy reduces loss of correction following chevron osteotomy. This has been particularly noticeable in the difference in the HVA between the first and last radiographic follow-up visit, as well as in the rates of recurrence (HVA > 20° and IMA > 10°) both in our study and in studies by other authors [19, 20]. Kaufman et al. evaluated the effect of additional Akin osteotomy in hallux valgus deformity correction via scarf osteotomy and achieved a similarly evident reduction in recurrence rates with the use of the additional technique (1.6% in comparison with 14.7%) [38]. Therefore, performing additional Akin osteotomy should be considered intraoperatively if the first metatarsal osteotomy does not produce adequate deformity correction. One biomechanical argument for the use of Akin osteotomy seems to be the change in the forces transferred via the extensor hallucis longus muscle, whose insertion is on the dorsal aspect of the distal phalanx of the hallux. Akin osteotomy shifts the extensor hallucis longus muscle insertion medially, with the resulting loss of the muscle’s abductor function, which is likely responsible for the lower number of forefoot deformity recurrences. The procedure involving extensor hallucis longus muscle insertion has been already presented as a technique for hallux valgus reconstruction surgery that ensures reduced recurrence rates [39].

The routine preparation for hallux valgus surgery should include measuring the IPA value. Further meta-analyses and studies in larger patient populations may contribute to developing a treatment algorithm and establishing specific angle values that would determine when Akin osteotomy should be performed. One study by Schilde et al., involving patients with an IPA of over 10°, compared the use of an additional Akin osteotomy performed via an open and minimally invasive approach [34]. Our study, like the study by Lechler et al., demonstrated an increase in IPA values from 6.9° to 8.2° in the chevron osteotomy group, whereas the additional use of Akin osteotomy helped achieve a decrease in this angle from 10.6° to 6.5° [20].

Our study data showed comparable rates of all types of complications in both groups. Lechler reported no need for revision surgery in either group [20]. Like other authors, we did not observe any cases of first metatarsal head necrosis or nonunion [20, 31, 36].

There have been no studies assessing adjacent-joint arthritis after hallux valgus correction surgery, particularly following combined distal chevron and proximal phalanx surgery. The long-term follow-up in our study showed no significant differences in the arthritis rates in any of the evaluated joints in either study group. This suggests good radiographic outcomes and a lack of negative effects of Akin osteotomy on the development of adjacent-joint arthritis, which makes Akin osteotomy a recommendable procedure.

In this study we assessed radiographic outcomes; however, we are planning subsequent studies to compare clinical, functional, and biomechanical outcomes in patients undergoing hallux valgus deformity correction with chevron and Akin osteotomy. Future studies should include random control studies and larger sample sizes.

The strengths of our study include a relatively large number of evaluated procedures, similar patient age in both study groups, one of only three surgeons conducting the procedures, a similar protocol of lateral and medial tissue release, and a long follow-up period. One limitation of this study is its retrospective nature, although many other studies were also retrospective analyses [11, 19, 23, 31–34].

## Conclusions

The additional use of Akin osteotomy alongside chevron osteotomy has no adverse effect on the correction of either HVA or IMA or on the complication rates.

Combined chevron and Akin osteotomy reduces the risk of HVA and IPA increase at long-term follow-up.

The use of additional Akin osteotomy does not increase the risk of adjacent-joint arthritis.

The use of Akin osteotomy in combination with chevron osteotomy should be indicated in the treatment of hallux valgus deformity.
